# The Current State of the Protected *Apis mellifera mellifera* Population in Russia: Hybridization and Nosematosis

**DOI:** 10.3390/ani11102892

**Published:** 2021-10-04

**Authors:** Milyausha Kaskinova, Elena Saltykova, Alexander Poskryakov, Alexey Nikolenko, Luisa Gaifullina

**Affiliations:** Ufa Federal Research Center, Institute of Biochemistry and Genetics, Russian Academy of Sciences, 450054 Ufa, Russia; saltykova-e@yandex.ru (E.S.); possash@yandex.ru (A.P.); a-nikolenko@yandex.ru (A.N.); lurim78@mail.ru (L.G.)

**Keywords:** *Apis mellifera*, nosematosis, Nosema apis, Nosema cerana, hybridization

## Abstract

**Simple Summary:**

The Southern Ural Mountains are a habitat for one of the remaining populations of the dark forest bees *Apis mellifera mellifera*. Using molecular genetic methods, we have established that there are processes of hybridization of this population with subspecies from the evolutionary lineage C. In addition, some colonies are affected by nosematosis. Therefore, it is necessary to take urgent measures to preserve this population.

**Abstract:**

The Southern Urals of Russia are the habitat of one of the surviving populations of the dark forest bee—the Burzyan population of *Apis mellifera mellifera*. In this study, we present the results of the subspecies identification of bee colonies in the Altyn-Solok Nature Reserve in the Southern Ural Mountains using the intergenic mtDNA COI-COII locus and the assessment of the prevalence of nosematosis. Analysis of the mtDNA *COI-COII* intergenic locus in the studied sample showed that 30.4% of the colonies belong to the lineage C. The PCR diagnostics of nosematosis in 92 colonies selected from different sectors of the Altyn-Solok Nature Reserve showed that about half of the analyzed colonies were infected with *Nosema apis*. *Nosema ceranae* was found in eight colonies. Both of these factors can lead to the extinction of this population of the dark forest bee.

## 1. Introduction

A decrease in the number of bee populations is observed worldwide [1,2]; this is a result of the action of various factors, including diseases [3,4,5] and hybridization [6,7]. One of the dangerous diseases of bees is nosematosis—causing damage to the intestines of adult bees with microsporidia *Nosema* sp. [8]. *Nosema* sp. possess only residual mitochondrial organelles and absorb ATP from the host’s cellular environment, causing energy and oxidative depletion in the bees [4,9]. Intestinal degeneration and an increase in the amount of food to compensate for the increased energy costs cause a high mortality rate in bee colonies affected by nosematosis under the conditions of a long winter period and limited food resources. The European honeybee *Apis mellifera* has two *Nosema* species: *N. apis* and *N. ceranae*. The original host of *N. ceranae* is the Asian bee *Apis cerana* [10]. It is suggested that the decline in the population of honeybees that has occurred in recent decades is associated with *N. ceranae* ([8,11,12,13,14]. *N. ceranae* is a more virulent pathogen than *N. apis* [15]. *N. apis*, unlike the invasive species *N. ceranae*, has a long co-evolutionary history with *A. mellifera* [15,16]. The first infestation of European bees with *N. ceranae* was recorded in 2005 in Spain [17]. Then, it spread over Europe [8], America [12], Australia [13], and North Africa [18]. Two methods have been developed to identify *Nosema* sp.—microscopic and molecular [19]. Microscopic techniques are relatively inexpensive and simple. Even though the morphology of the spores of the two *Nosema* species is different [20], it is difficult to differentiate these species at a low level of infection or the vegetative stage of pathogen development. Therefore, it became necessary to develop another diagnostic method. PCR analysis has become an important addition to microscopy, as it allows one to identify the pathogen in the vegetative stage of development [8,12,21].

The problem of preserving the gene pool of different subspecies of the honeybee was raised because hybridization leads to the loss of the gene association characteristics of each specific subspecies and ecotype which are responsible for adaptability and ecological plasticity [6,7,22,23,24]. The import of packages of bee colonies belonging to the evolutionary lineage C, which includes the two most common subspecies in commercial beekeeping—*A. m. ligustica* and *A. m. carnica*, led to the fragmentation of the *A. m. mellifera* range. The problem of preserving populations of *A. m. mellifera* is relevant for most European countries [25,26,27]. So, in 1964, an association was created, now known as BIBBA (Bee Improvers and Bee Breeders Association), whose goals were to preserve, restore and study the dark forest bee, adapted to habitats in Great Britain and Ireland [28]. In Sweden, in 1990, a project was organized to protect local populations of the dark forest bee [29]. To restore the gene pool of the dark forest bee, it is necessary to search and further monitor the surviving populations.

Part of the territory of the Southern Urals (Republic of Bashkortostan, Russia) is the habitat of the Burzyan population of *A. m. mellifera*. Since 1958, this population has been under the protection of the Shulgan-Tash Nature Reserve. Since 1997, the preservation of the Burzyan bee population has been the main task of the Altyn Solok reserve. The winter of 2016–2017 had an extremely negative effect on the number of wild-hive colonies in the protected areas; this prompted us to conduct an assessment of the situation on the Altyn Solok Nature Reserve. We set the following task—to investigate wild and man-kept *A. m. mellifera* colonies on the reserve territory for the presence of nosematosis and to determine whether hybridization processes occur in this population.

## 2. Materials and Methods

Sampling was carried out in 27 sectors of the reserve and one settlement on the border with the reserve. A total of 92 colonies were analyzed. Initially, we selected bees from 58 colonies (from 1 to 5 colonies from 27 reserve sectors and one settlement). To establish the situation within one sector, 34 additional colonies were sampled. DNA isolation was carried out from the middle intestines of worker bees (5 bees per colony) using GeneJET™ (Thermo Fisher Scientific, Waltham, MA, USA). The DNA was eluted in a final volume of 100 µL and the extracts were stored at −30 °C until used as a template in the PCR.

**Figure 1:**
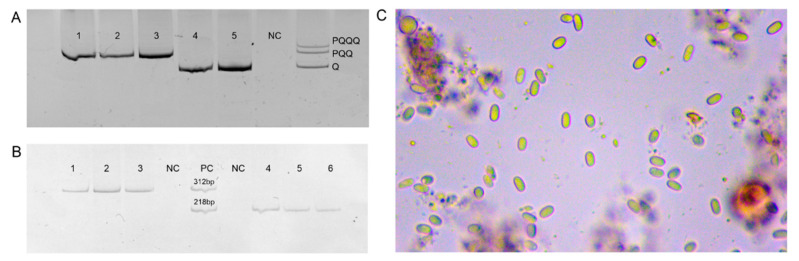
Gel images of PCR products: (**A**)—mtDNA *COI-COII* intergenic locus; (**B**)—16S rRNA gene fragment of *N. apis* (1–3) and *N. ceranae* (4–6); NC—negative control; PC—positive control; (**C**)—*Nosema* spore (400×) in macerated abdomen suspension of adult honeybees.

The belonging of bees to the evolutionary lineage M or C was determined using the analysis of the mtDNA *COI-COII* intergenic locus (F-GGCAGAATAAGTGCATTG and R- GGTCATCAATGATATTG) [30]. Analysis of the intergenic mtDNA *COI-COII* locus is one of the simple and reliable methods for differentiating the evolutionary lineages of M and C [31]. Allelic variants P(Q)_1−n_ are markers of the origin of the bees from *A. m. mellifera*, variant Q—from subspecies from the evolutionary lineage C on the maternal line. The PCR mix included 17 μL distilled water; 2 μL 10-fold Taq buffer (25 mM Mg^2+^); 0.4 μL dNTP (10 μM); 0.6 μL F and R primers (2 OE); and 0.3 μL Taq polymerase. The PCR mode: 5 min at 94 °C; then, 30 cycles with denaturation for 30 s at 94 °C; annealing for 30 s at 49 °C; elongation for 60 s at 72 °C; and final elongation for 7 min at 72 °C. The gels (8% polyacrylamide) were stained with ethidium bromide and visualized using Gel Doc XR+ (Figure 1A).

To differentiate between the two *Nosema* species, two sets of primers were used: F-5′-gggggcatgtctttgacgtactatgta-3′ and R-5′-ggggggcgtttaaaatggaaacaactatg-3′ [21], and F-5′-ccattgccggataagagagt-3′ and R-5′-cacgcattgctgcatcattgac-3′ [12] for *N. apis*; F-5′-cggcgacgatgtgatatgaaaatattaa-3′ and R-5′-cccggtcattctcaaacaaaaaaccg-3′ [21], and F-5′-cggataaaagagtccgttacc-3′ and R-5′-tgagcagggttctagggat-3′ [12] for *N. ceranae*. The PCR mix was the same as described above. The PCR mode: 5 min at 94 °C; then 30 cycles with denaturation for 30 s at 94 °C; annealing for 30 s at 48 °C; elongation for 60 s at 72 °C; and final elongation for 7 min at 72 °C. Amplification products were visualized in 8% polyacrylamide (Figure 1B). A DNA sample from a colony with a high level of infection with nosematosis, confirmed by microscopy, was used as a positive control.

Microscopic examination of intestinal contents was carried out individually in 10 bees from each colony. The intestine of one bee was rubbed with 1 mL of distilled water, a drop of the resulting suspension was examined under a microscope at a magnification of 400× using a Biomed microscope (Russia). 

## 3. Results

Analysis of the polymorphism of the *COI-COII* locus showed that 30.4% of the analyzed colonies belong to the evolutionary lineage C (allelic variant Q, Table 1). Nosematosis was found in 50 of the 92 colonies (42 positives for *N. apis* and 8—for *N. ceranae*). Microscopic examination of the contents of the middle intestines of the bees from these colonies showed the presence of spores only in those colonies where the amount of amplification product was large (Figure 1C).

Our spot sampling showed that about half of the colonies suffer from nosematosis. In the sector where the largest number of samples was taken (39 colonies), *N. apis* was found in 33 colonies and *N. ceranae* in one colony. 

**Figure 2:**
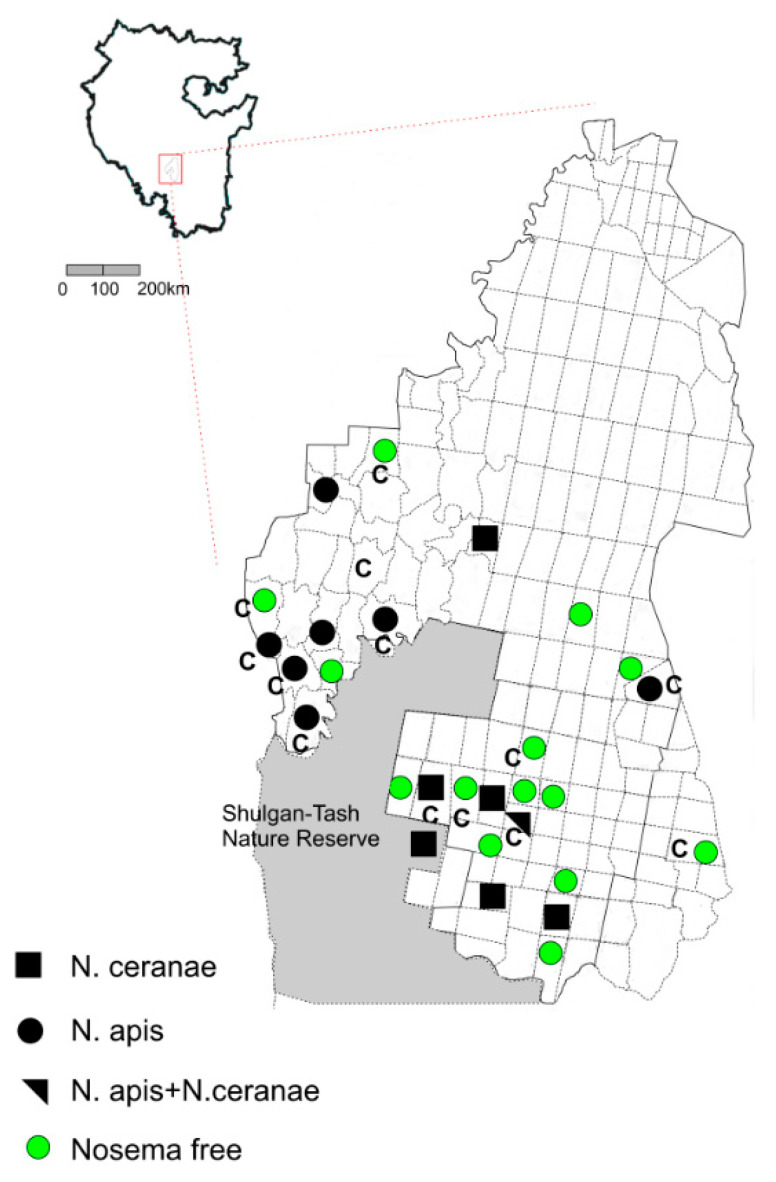
The geographic location of colonies affected by nosematosis on the territory of the Altyn-Solok Nature Reserve. C—sectors where the allelic variant Q of the locus *COI-COII* mtDNA was found in bee colonies. The part of the Shulgan-Tash Nature Reserve is in light gray.

*N. apis* was found in 8 out of 28 sectors and *N. ceranae* in 7 sectors. Coinfection with *N. apis* and *N. ceranae* was found only in one sector of the reserve, but not within one colony. Figure 2 shows that *N. ceranae* prevailed in the southern part of the Altyn Solok Nature Reserve. The part of Shulgan-Tash Nature Reserve is located between these samples. 

## 4. Discussion

The Burzyan population is one of the native populations of the dark forest bee *A. m. mellifera* in Russia. The first studies of the genetic diversity of the Burzyan population of the dark forest bee *A. m. mellifera*, using the intergenic *COI-COII* locus, began in the 1990s in connection with the massive introduction of the *A. m. caucasica* and *A. m. carnica* subspecies to the Republic of Bashkortostan [32]. At that time, the introgression of the lineage C subspecies was not registered. Introgression at the mtDNA level in the Burzyan population was detected for the first time in 2004—two colonies out of 66 had allelic variant Q [33,34]. Thus, over 15 years, the level of introgression has increased 10 times. The appearance of bee colonies of the subspecies from lineage C in the protected area is possible in two ways. First, by the arrival of swarms from the adjacent territories where there are no restrictions on the import of bee packages. Second, by illegal importation of such colonies by nomadic beekeepers. 

Nosematosis was found both in colonies belonging to the evolutionary lineage M and C (Figure 2). As the studied sample is very small, we did not conduct a test to find an association between belonging to the evolutionary lineages M/C and the prevalence of nosematosis. 

Restrictions on the import of bees did not protect the Burzyan population of *A. m. mellifera* from hybridization. In particular, this was due to the lack of constant genetic monitoring of the purity of the bee colonies imported by the local and nomadic beekeepers. The facts of the C lineage introgression at the mitochondrial genome level in the protected area of the Burzyansky district were previously recorded only sporadically. According to the mitochondrial DNA *COI-COII* locus analysis, 30.4% of the colonies in the Altyn Solok Nature Reserve belong to the evolutionary lineage C. Considering that almost half of the studied colonies are infected with nosematosis, this poses a threat to this population. 

## 5. Conclusions

In this study, we present the results of the subspecies identification of bee colonies in the Altyn-Solok Nature Reserve in the Southern Ural Mountains and the assessment of the prevalence of nosematosis. We found that more than half of the bee colonies are affected by nosematosis. In addition, 30.4% of the colonies belong to the lineage C. Both of these factors can lead this population to extinction.

The methods of preserving local populations of honeybees include the creation of protective zones, the monitoring of honeybee subspecies, and working with beekeepers, involving them in breeding local honeybees. All these methods have been applied to the Burzyan bee population since its discovery. 

In 1958, the Shulgan-Tash Nature Reserve was organized. The basis for the organization of the reserve was the habitation in this region of the nucleus of a purebred local population of the honeybee—the Burzyan bee. In 1997, a regional Altyn Solok Nature Reserve was created in order to protect the Burzyan population of *A. m. mellifera*. In 2012, these nature reserves were added to the UNESCO Biosphere Reserve “Bashkir Ural” [35]. It is forbidden to bring bees to these protected areas. The total area of these protected areas is small (225 km^2^ for the Shulgan-Tash Nature Reserve and 899 km^2^ for the Altyn Solok Nature Reserve, i.e., about 25% of the territory of the Burzyansky district). Nevertheless, the results of our studies of this population [32,33,34] and the reports of beekeepers, who repeatedly complain to the government about nomadic beekeepers from other regions and trucks selling bees of unknown origin on the border with the reserve, indicate the ongoing hybridization processes. Therefore, it is necessary to prohibit the import of bees not only to the protected areas but to the entire territory of the Burzyan district. In Norway, only *A. m. mellifera* is allowed to be kept in an area of approximately 3,500 km^2^, and approximately the same areas are set aside for *A. m. carnica* [36]. Similar tactics of beekeeping can be introduced in the Southern Urals—to highlight the territories where *A. m. mellifera* or *A. m. carnica* are and to breed them within the designated area. 

As for the monitoring of subspecies, since 1999 our laboratory has been working with this population of bees and confirms its belonging to the evolutionary line M [32]. Since then, we have seen a gradual increase in hybridization in this bee population [34]. Consequently, monitoring does not always bring about the desired results as we cannot control whether our data were used by the beekeepers and whether the queens were replaced in the hybrid colonies. The beekeepers of the Burzyansky district are interested in preserving the local population of honeybees; this is inherent in their mentality. However, in order to compensate for the loss of the bee colonies, the beekeepers are forced to buy bee colonies of unknown origin. Often these are packages with subspecies from the C evolutionary lineage as they are more readily available. However, this is not the only problem. There are a lot of linden forests in the territory of the Burzyansky district. This attracts a large number of nomadic beekeepers from neighboring districts and even regions.

A way out of this situation is the creation of centers for breeding dark forest bees in order to create an alternative to bee packages of unknown origin. To create such a center, the original material is needed. We are currently dealing with this problem—we are looking for the remaining reserves of the dark forest bee and, in cooperation with beekeepers, we intend to try to increase their number. It is obvious that the analysis of the mitochondrial DNA is not enough to assess the level of introgression. For a more complete understanding of the genetic structure of the population, we intend to use the analysis of the SSR loci of nuclear DNA in our future studies of this population.

## Figures and Tables

**Table 1:** Distribution of nosematosis in colonies belonging to the evolutionary lineages M (allelic variant PQQ of the *COI-COII*) and C (allelic variant Q) in the territory of the Altyn Solok reserve.

Evolutionary Lineage	N. of Colonies	N. of Colonies Infested with *N. apis* (%)	N. of Colonies Infested with *N. ceranae* (%)
M	64	31 (48.4%)	7 (10.9%)
C	28	11 (39.3%)	1 (3.6%)
In total:	92	42 (45.7%)	8 (8.7%)

## References

[1] Goulson D., Nicholls E., Botias C., Rotheray E.L. (2015). Bee declines driven by combined stress from parasites, pesticides, and lack of flowers. Science.

[2] Theisen-Jones H., Bienefeld K. (2016). The Asian honey bee (*Apis cerana*) is significantly in decline. Bee World.

[3] Brown M.J.F., Paxton R.J. (2009). The conservation of bees: A global perspective. Apidologie.

[4] Alaux C., Brunet J.L., Dussaubat C., Mondet F., Tchamitchan S., Cousin M., Brillard J., Baldy A., Belzunces L.P., Le Conte Y. (2010). Interactions between Nosema microspores and a neonicotinoid weaken honeybees (*Apis mellifera*). Environ. Microbiol..

[5] Bromenshenk J.J., Henderson C.B., Wick C.H., Stanford M.F., Zulich A.W., Jabbour R.E., Deshpande S.V., McCubbin P.E., Seccomb R.A., Welch P.M. (2010). Iridovirus and Microsporidian Linked to Honey Bee Colony Decline. PLoS ONE.

[6] Jensen A.B., Palmer K.A., Boomsma J.J., Pedersen B.V. (2005). Varying degrees of *Apis mellifera* ligustica introgression in protected populations of the black honeybee, *Apis mellifera mellifera*, in northwest Europe. Mol. Ecol..

[7] Themudo G.E., Rey-Iglesia A., Tascon L.R., Jensen A.B., da Fonseca R.R., Campos P.F. (2020). Declining genetic diversity of European honeybees along the twentieth century. Sci. Rep..

[8] Klee J., Besana A.M., Genersch E., Gisder S., Nanetti A., Tam D.Q., Chinh T.X., Puerta F., Ruz J.M., Kryger P. (2007). Widespread dispersal of the microsporidian *Nosema ceranae*, an emergent pathogen of the western honey bee, *Apis mellifera*. J. Invertebr. Pathol..

[9] Dussaubat C., Brunet J.L., Higes M., Colbourne J.K., Lopez J., Choi J.H., Martin-Hernandez R., Botias C., Cousin M., McDonnell C. (2012). Gut Pathology and Responses to the Microsporidium *Nosema ceranae* in the Honey Bee *Apis mellifera*. PLoS ONE.

[10] Fries I., Feng F., da Silva A., Slemenda S.B., Pieniazek N.J. (1996). *Nosema ceranae* n sp (*Microspora, Nosematidae*), morphological and molecular characterization of a microsporidian parasite of the Asian honey bee *Apis cerana* (*Hymenoptera*, *Apidae*). Eur. J. Protistol..

[11] Chauzat M.P., Higes M., Martin-Hernandez R., Meana A., Cougoule N., Faucon J.P. (2007). Presence of *Nosema ceranae* in French honey bee colonies. J. Apic. Res..

[12] Chen Y., Evans J.D., Smith I.B., Pettis J.S. (2008). *Nosema ceranae* is a long-present and wide-spread microsporidian infection of the European honey bee (*Apis mellifera*) in the United States. J. Invertebr. Pathol..

[13] Giersch T., Berg T., Galea F., Hornitzky M. (2009). *Nosema ceranae* infects honey bees (*Apis mellifera*) and contaminates honey in Australia. Apidologie.

[14] Sharma D., Katna S., Sharma R., Rana B.S., Sharma H.K., Bhardwaj V., Chauhan A. (2019). First detection of *Nosema ceranae* infecting *Apis mellifera* in India. J. Apic. Sci..

[15] Paxton R.J., Klee J., Korpela S., Fries I. (2007). *Nosema ceranae* has infected *Apis mellifera* in Europe since at least 1998 and may be more virulent than Nosema apis. Apidologie.

[16] Williams G.R., Shutler D., Burgher-MacLellan K.L., Rogers R.E.L. (2014). Infra-Population and -Community Dynamics of the Parasites Nosema apis and *Nosema ceranae*, and Consequences for Honey Bee (*Apis mellifera*) Hosts. PLoS ONE.

[17] Higes M., Martin R., Meana A. (2006). *Nosema ceranae*, a new microsporidian parasite in honeybees in Europe. J. Invertebr. Pathol..

[18] Higes M., Martin-Hernandez R., Garrido-Bailon E., Botias C., Meana A. (2009). The presence of *Nosema ceranae* (*Microsporidia*) in North African honey bees (*Apis mellifera intermissa*). J. Apic. Res..

[19] Fries I., Chauzat M.P., Chen Y.P., Doublet V., Genersch E., Gisder S., Higes M., McMahon D.P., Martin-Hernandez R., Natsopoulou M. (2013). Standard methods for Nosema research. J. Apic. Res..

[20] Ptaszynska A.A., Borsuk G., Mulenko W., Demetraki-Paleolog J. (2014). Differentiation of Nosema apis and *Nosema ceranae* spores under Scanning Electron Microscopy (SEM). J. Apic. Res..

[21] Martin-Hernandez R., Meana A., Prieto L., Salvador A.M., Garrido-Bailon E., Higes M. (2007). Outcome of colonization of *Apis mellifera* by *Nosema ceranae*. Appl. Environ. Microbiol..

[22] Chapman N.C., Harpur B.A., Lim J., Rinderer T.E., Allsopp M.H., Zayed A., Oldroyd B.P. (2016). Hybrid origins of Australian honeybees (*Apis mellifera*). Apidologie.

[23] Oldroyd B.P., Cornuet J.M., Rowe D., Rinderer T.E., Crozier R.H. (1995). Racial admixture of *Apis mellifera* in Tasmania, Australia—Similarities and differences with natural hybrid zones in Europe. Heredity.

[24] Oleksa A., Chybicki I., Tofilski A., Burczyk J. (2011). Nuclear and mitochondrial patterns of introgression into native dark bees (*Apis mellifera mellifera*) in Poland. J. Apic. Res..

[25] Hassett J., Browne K.A., McCormack G.P., Moore E., Soland G., Geary M. (2018). A significant pure population of the dark European honey bee (*Apis mellifera mellifera*) remains in Ireland. J. Apic. Res..

[26] Parejo M., Wragg D., Gauthier L., Vignal A., Neumann P., Neuditschko M. (2016). Using Whole-Genome Sequence Information to Foster Conservation Efforts for the European Dark Honey Bee, *Apis mellifera mellifera*. Front. Ecol. Evol..

[27] Pinto M.A., Henriques D., Chavez-Galarza J., Kryger P., Garnery L., van der Zee R., Dahle B., Soland-Reckeweg G., de la Rua P., Dall’Olio R. (2014). Genetic integrity of the Dark European honey bee (*Apis mellifera mellifera*) from protected populations: A genome-wide assessment using SNPs and mtDNA sequence data. J. Apic. Res..

[28] Bee Improvement & Bee Breeders Association. https://bibba.com/.

[29] Föreningen Nordbi. https://www.nordbi.se/.

[30] Evans J.D., Schwarz R.S., Chen Y.P., Budge G., Cornman R.S., De la Rua P., de Miranda J.R., Foret S., Foster L., Gauthier L. (2013). Standard methods for molecular research in *Apis mellifera*. J. Apic. Res..

[31] Bertrand B., Alburaki M., Legout H., Moulin S., Mougel F., Garnery L. (2015). MtDNA COI-COII marker and drone congregation area: An efficient method to establish and monitor honeybee (*Apis mellifera* L.) conservation centres. Mol. Ecol. Resour..

[32] Nikolenko A.G., Poskryakov A.V. (2002). Polymorphism of locus COI-COII of mitochondrial DNA in the honey bee *Apis mellifera* L. from the Southern Ural Region. Russ. J. Genet..

[33] Il’yasov R.A., Petukhov A.V., Poskryakov A.V., Nikolenko A.G. (2007). Local honeybee (*Apis mellifera mellifera* L.) populations in the Urals. Russ. J. Genet..

[34] Kaskinova M.D., Gataullin A.R., Khasanov M.V., Ilyasov R.A., Kwon H.W., Nikolenko A.G. (2018). The purebredness estimation of *Apis mellifera mellifera* L. population in the Altyn-Solok conservancy area. Proc. RAS Ufa Sci. Cent..

[35] UNESCO Tentative Lists. https://whc.unesco.org/en/tentativelists/5666/.

[36] Bouga M., Alaux C., Bienkowska M., Buchler R., Carreck N.L., Cauia E., Chlebo R., Dahle B., Dall’Olio R., De la Rua P. (2011). A review of methods for discrimination of honey bee populations as applied to European beekeeping. J. Apic. Res..

